# Grafting on resistant rootstocks alleviates salt stress on tomato by enhancing morpho-physiological and biochemical traits

**DOI:** 10.1007/s00709-026-02222-w

**Published:** 2026-06-01

**Authors:** Md Sarowar Alam, Mark Tester, Gabriele Fiene, Gordon Wellman, Magdi Ali Ahmed Mousa

**Affiliations:** 1 Department of Agriculture, Faculty of Environment Science, King Abdulaziz University, Jeddah, 21589 Saudi Arabia; 2 Regional Agricultural Research Station, Bangladesh Agricultural Research Institute (BARI), Akbarpur, Moulvibazar 3210 Bangladesh; 3 Biological and Environmental Sciences and Engineering Division, King Abdullah University of Science and Technology (KAUST), Thuwal, 23955 Saudi Arabia; 4 Department of Vegetables, Faculty of Agriculture, Assiut University, Assiut, Egypt

**Keywords:** Salinity, Rootstocks, Ions, Antioxidants, Grafting

## Abstract

Salinity is a global threat to crop production, reducing yields and jeopardizing food and nutrition security. This study was performed to evaluate the salinity tolerance of high-yielding commercial tomato ‘Super Sweet F_1_’(SS), which was self-grafted, or cross-grafted with salt-tolerant rootstocks ‘Maxifort F_1_’ (MF), ‘Pimp-M021’(PM), and ‘Ramsi’ (RM). The plants were evaluated at low (18.2 mM NaCl) and high-water salinity (150 mM NaCl). The cross-grafted plants SS/MF and SS/PM showed similar responses under high salinity with increased leaf chlorophyll concentration, relative water content (RWC), membrane stability index (MSI), Na^+^ and K^+^ concentration, electrolyte leakage (EL), stomatal conductance, fruit number, and weight, when compared to non-grafted and self-grafted plants. Due to the salt stress, elevated activities of proline, total phenols (TPC), total flavonoids (TFC), polyphenol oxidase (PPO; EC 1.14.18.1), peroxidase (POD; EC 1.11.1.7), catalase (CAT; EC 1.11.1.6), and superoxide dismutase (SOD; EC 1.15.1.1) were also measured in the leaves of SS/MF, SS/PM, and SS/RM in comparison with SS and SS/SS. The principal component biplot analysis confirmed that the grafted tomatoes, SS/MF and SS/PM, showed improved performance under salt stress compared with non-grafted, self-grafted, and cross-grafted SS/RM plants. This study revealed that Pimp-M021’(PM) could be a promising candidate beside the commercial Maxifort F_1_ (MF) rootstock for tomato under salt stress conditions. Our findings demonstrated the positive effects of salt-tolerant rootstocks on tomato growth, yield, and fruit characteristics, and this could herald a sustainable tomato production practice in salinity-prone areas worldwide.

## Introduction

Salinity is one of the most significant abiotic stresses affecting agriculture, causing growth reduction, physiological abnormalities, and significant yield losses of crops worldwide (Munns and Tester 2008; Tan et al. 2020; El-Banna et al. 2022; Shahzad et al. 2022; Semmoudi et al. 2025). About half of the global arable land will become saline soils by 2050, because of the negative effects of climate change, irrigation with saline water, and intensive agricultural practices (Tuteja 2007; Ondrasek et al. 2022). Based on the United Nations’ annual reports, about 1.5 million hectares of global farmland are lost annually due to soil salinization (FAO, 2022). Salt accumulation in plant tissues leads to adverse changes in plant morphology, physiology, and biochemistry, resulting in limited growth and yield losses (Hatam et al. 2020; El-Banna et al. 2022). Exposure to high salinity results in reduced plant growth, and the osmotic stress enhances the formation of smaller leaves with a dark bluish-green appearance. More precisely, exposure to salt stress encourages the accumulation of Na^+^ and Cl^+,^ and nutritional imbalance (Gharsallah et al. 2020; Hatam et al. 2020). There are three main categories of response mechanisms of plants to salinity stresses: (1) osmotic tolerance, the plants keep slowing their growth and development through reduced water loss, slow leaf expansion processes, and keep stomatal conductance processes at minimum levels, (2) ion exclusion, prevent plant roots to absorb high levels of Na^+^ ions to keep at a lower level than its toxic concentrations in the shoots and leaves, and (3) tissue tolerance, remove Na^+^ ions from the cell cytosol and isolate it in the vacuoles to protect the plant cells from negative effects of Na^+^ toxicity (Munns and Tester 2008; Bonarota et al. 2022; Sarkar et al. 2026).

Tomato (*Solanum lycopersicum* L.) is a member of the Solanaceae family, produced for fresh consumption and processed foods worldwide (Campestrini et al. 2019; Li et al. 2021). The fruit contain 95% water, 4% carbohydrates, as well as less than 1% of proteins and fats. It is also rich in many nutrients, essential elements, pigments (ß-carotenoids and lycopene), vitamins (C and E), and phenolic compounds, which are considered beneficial for human health (Tomlinson et al. 2017; Vats et al. 2020). Salinity is a major threat to sustainable tomato production as it renders a considerable decline in growth and productivity (Foolad 2004; Alqardaeai et al. 2024; Sarkar et al. 2026). Most tomato cultivars are considered moderately salt-sensitive, and salinity affects the tomato life cycle from seed germination through the reproductive stages (Bonarota et al. 2022; Van Zelm et al. 2020). Salt stress influences all stages of the tomato life cycle, including delayed seed germination (Tanveer et al. 2020), reduction of photosynthetic pigments concentration, root and shoot length, and dry weight, as well as leaf area and number (Rahman et al. 2018; Maeda et al. 2020). Additionally, salinity stress enhances the degradation of photosynthesis and photorespiration, and reduces transpiration, stomatal activity, and leaf water status (Bonarota et al. 2025). Excess salt disturbs the ion uptake channel and enhances the accumulation of harmful ions with the suppression of metabolically crucial compounds (Maeda et al. 2020; Maqbool et al. 2020; Munns et al. 2020). Moreover, disturbance of biochemical and hormonal activities in plants (Moles et al. 2019) and, finally, curtails the fruit yield (Costan et al. 2020; Maeda et al. 2020). Salinity exerts a detrimental effect on the photosynthesis of plants by a combined effect of enzymatic activities inhibition, such as ribulose-1,5-bisphosphate carboxylase/oxygenase (Rubisco) and phosphoenolpyruvate (PEP) carboxylase, responsible for chlorophyll biosynthesis and the degradation of chlorophyll by the activation of chlorophyllase (Zhu 2001; Santos 2004). Accumulation of specific osmolytes in stressed plants plays a significant role in cellular osmotic adjustment (Shahid et al. 2020). Proline is a major osmolyte generated in stressed plants, acts as a low molecular weight chaperon, ROS scavenger, gene expression regulator, and metabolic process as a signaling molecule (Ashraf and Harris 2004; Szabados and Savouré 2010; Slama et al. 2015). Salinity stress induces oxidative responses by generating reactive oxygen species (ROS), and plants combat these stresses through the activity of antioxidant defense systems, including non-enzymatic antioxidants such as phenols and flavonoids, and enzymatic antioxidants such as PPO, POD, CAT, and SOD (El-Banna et al. 2022; Semmoudi et al. 2025).

The negative effects of salinity on the growth and yield of tomatoes can be alleviated by different strategies such as seed priming, genetic modification, applying bio-stimulants, and grafting on resistant rootstocks (Farouk et al. 2020; Farouk and Al-Huqail 2022). Plant breeding and biotechnological interventions have been going on over the years to improve the salinity tolerance in tomato, but this is very difficult due to the complex genetic nature of the salinity tolerance genes and their expression in plants. So, grafting is a sustainable method to improve growth and yield under salt stress by combining productive scions with tolerant rootstocks (Coban et al. 2020; Parthasarathi et al. 2021). Grafting is routinely practiced for managing soil-borne diseases and nematodes (Hamdi et al. 2009; Owusu et al. 2016; Sarkar et al. 2026). Grafting of commercial hybrid tomatoes onto salt-tolerant rootstocks could enhance the growth, yield, and fruit quality under normal and stress conditions by limiting the translocation of harmful ions to the shoots, maintaining photosynthetic activities, and absorption of adequate moisture and essential elements (Savvas et al. 2017; Soare et al. 2018; Aydın, 2024). The rootstock can be an F_1_ hybrid, wild accessions/strains/lines, or heirloom cultivar (Zeist et al. 2023; Martínez et al. 2024; Muslu et al. 2024). Generally, a desirable rootstock has a strong root system, vigorous growth, and resistance to pests, diseases, and abiotic stresses (Singh et al. 2017; Grieneisen et al. 2018; Bonarota et al. 2022). For improving growth and yield under salt stress, the plants should be grafted on rootstocks with ion exclusion capabilities to prevent the absorption of higher concentrations of Na^+^ and Cl^+^ and subsequently, avoid the accumulation of toxic levels of Na^+^ and Cl^+^ in the leaves and shoots, generate osmolytes, and upregulate the antioxidant defence system (Savvas et al. 2011; Albacete et al. 2015; Asins et al. 2015; Bonarota et al. 2022; Alqardaeai et al. 2024). However, there are few studies on the evaluation of the rootstocks for enhanced salinity tolerance in tomato and in-depth physiological and biochemical mechanisms underlying the salt tolerance. The hypothesis was that grafting tomato onto different salt-tolerant rootstocks would significantly increase the plant physiological and biochemical parameters, fruit yield, and quality in tomato. The present study was conducted to evaluate the long-term response of grafted tomatoes to irrigation water salinity under greenhouse conditions based on morphological, physiological, and biochemical analysis.

## Materials and methods

### Planting materials

The tomato commercial hybrid ‘Super Sweet F_1_’ (SS) used as the scion was obtained from the agricultural market in Jeddah, Saudi Arabia. Seeds of the widely used interspecific hybrid rootstock ‘Maxifort F_1_’ (MF) were obtained from Johnny’s Selected Seeds company (955 Benton Ave, Winslow, ME 04901, USA). The wild *Solanum pimpinellifolium* accession ‘Pimp-M021’ (PM) was found as salt tolerant and recommended as a rootstock to improve salinity tolerance in tomato (Johansen et al. 2020). The ‘Ramsi (RM)’ tomato (*Solanum lycopersicum* L.) is a salt-tolerant landrace cultivar adapted to the Qatif oasis in the Eastern province of Saudi Arabia along the Arabian Gulf. Seeds of PM and RM were provided by Prof. Mark Tester (Centre for Desert Agriculture, KAUST, Saudi Arabia).

### Raising the tomato seedlings

Germination of tomato seedlings was performed in the controlled conditions in the core labs’ greenhouse. Seeds of the two rootstocks ‘MF’ and ‘RM’ were sown on 15 October 2019 in 50 mm coco coir pellets, while ‘PM’ was sown seven days earlier due to slower growth and a thinner stem diameter. To ensure a similar stem diameter for grafting to increase cambial union and graft compatibility, seeds of the tomato scion ‘SS’ were sown 4, 8, and 12 days after sowing the rootstocks’ seeds. Scion seeds were sown in large plastic trays with small holes in the bottom and filled with a mixture of peat moss and perlite (2:1) to ensure adequate drainage. Both the rootstocks and scion seedlings were grown in a greenhouse with day/night temperatures of 23°C/ 18°C, relative humidity of 70–75%, photoperiod of 16 hours.

### Grafting procedure

After 25–30 days of germination, when the third true leaves had emerged, the seedlings were well watered for 12–16 h before grafting with the splice grafting method. Before grafting, the working tables, grafting clips, and blades were surface sterilized using 5% sodium hypochlorite. Seedlings of the commercial tomato ‘SS’ were self-grafted (SS/SS) and grafted to the rootstocks: SS/MF, SS/PM, and SS/RM.

### Healing of grafted plants

Just after the grafting operation, the grafted plants were transferred to a healing chamber and healed under 98% humidity, 26 °C in complete darkness for 2 days. The humidity level was achieved at 98% by a humidifier and misting the chamber with water, and the grafts were kept for 10–14 days at 20˚C during the day and 16˚C at night temperature and humidity of > 95%. After keeping for 14 days in the healing chamber, the grafted plants were transferred on the 15th day from the healing chamber to the greenhouse and subjected to an acclimatization protocol.

### Acclimatization of successful grafts

The plants were acclimatized by exposing them to sunlight and ambient greenhouse temperature daily for seven days. The greenhouse temperature was adjusted at 24.0–29.5 °C, and humidity ranged from 78.5 to 100% for 10 days. The plants were kept hydrated by applying water to the base of the rootstock. At this stage, side shoots on the rootstocks were removed while those on the scion were kept, allowing for new growth. The grafted seedlings were transplanted in plastic pots filled with a medium composed of 3:1 peat and perlite. Plants were routinely irrigated and fertilized with an N: P:K (20:20:20) nutrients solutions (Atlantic Agricola, Spain).

### Greenhouse experiment

#### Experimental design

The greenhouse experiment was conducted from 15 October 2019 to 15 May 2020 in the plant growth core laboratory located at KAUST, Thuwal, Saudi Arabia. Grafted seedlings combinations SS/SS (self-grafted), SS/MF, SS/PM, and SS/RM (cross-grafted), and SS (non-grafted) seedlings were transferred to 20 cm diameter and 10 L volume plastic pots filled with coconut growing media connected with a hydroponic system for irrigation. The experiment was carried out in a split-plot design where the treatments (SS, SS/SS, SS/MF, SS/PM, and SS/RM) were distributed randomly following a completely randomized block design (RCBD) with three replications. There were five plants for each of the grafting combinations (SS, SS/SS, SS/MF, SS/PM, and SS/RM) and one seedling per pot. The salinity levels were in the main plots, and grafting combinations were in the sub-plots.

#### Salt stress application and nutrient management

After the establishment of the seedlings (one week of transplantation), the seedlings were subjected to salt stress treatments. Two levels of salinity: fresh water or low salinity as control (S1:18.2 mM NaCl) and salt stress treatment (S2: 150 mM NaCl) were applied with irrigation. To avoid shock to the seedlings, irrigation water salinity was gradually increased. We started with a water salinity with 50 mM NaCl on the first day of salt stress and gradually increased with 50 mM NaCl every second day until water salinity reached 150 mM NaCl, then the stress application was continued for four weeks, and then normal irrigation and nutrients supplementation consisted of total nitrogen (N) (20%), phosphorous pentoxide (P_2_O_5_) (20%), potassium oxide (K_2_O) (20%), iron (Fe) EDTA (0.060%), manganese (Mn) EDTA (0.050%), zinc (Zn) EDTA (0.012%), copper (Cu) EDTA (0.003%), boron (B) water soluble (0.020%), and molybdenum (Mo) water soluble (0.003%) (Atlantic Agricola, Spain) were applied until the end of the experiment. To prevent NaCl accumulation in the growing media, four times irrigation with extra freshwater with a nutrient solution was applied to the pots. To ensure a similar quality of stress imposition, the EC and pH values were monitored from the leached solution from the pots. To maintain Ca^2+^ activity and proper distribution of ions inside the plant body, a lower concentration (2 mM) of CaCl_2_ was applied with the stress treatment (Tester and Davenport 2003; Negrão et al. 2017).

### Measurements

#### Leaf photosynthetic pigments

The fully expanded leaf samples from all the grafted, self-grafted, and non-grafted combinations were collected at the 4th week of stress application from both levels of salinity treatments. The concentrations of chlorophyll a (*Chl*-a), chlorophyll b (*Chl*-b), total chlorophyll, and carotenoids were determined according to Arnon (1949), and spectrophotometrically measured using (UV/Visible Spectrophotometer-LKB-Biochrom-4050) according to Metzner et al. (1965).

#### Leaf inorganic ions concentration

The inorganic ions (Na^+^, Ca^2+^_,_ and K^+^) concentration in the dried leaves was determined according to (Jones et al. 1990). Leaf samples of non-grafted, self-grafted, and cross-grafted tomatoes were collected from both levels of stress-treated plants just after the completion of the 4th week, then oven-dried at 80 °C and ground using a mortar and pestle; 0.1 g of powder was then digested with 5 ml of nitric acid for 3 h. Then, the concentrations of Na^+^, K^+^ and Ca^2+^ were analysed by using Flame Photometer (Biobase BK-FP6450) (Dasgan et al. 2002).

#### Leaf Relative Water Content (LRWC)

LRWC was measured using leaflets of fully expanded leaves collected just after the fourth week of salt application from both levels of salinity treatments for four plants per treatment per replicate. A single leaf per plant was cut, and fresh weight (FW) was immediately measured. The leaflets were immersed in dd-H_2_O in petri dishes and incubated at room temperature for 4 h. The turgid weight (TW) of the leaflets was measured after being wiped to remove the water from the surface. The leaflet’s dry weight (DW) was measured after oven-drying of the leaflets at 70 °C for 24 h. Leaf relative water content was calculated using the following formula:

$$\%\mathrm{R}\mathrm{W}\mathrm{C}=\frac{(\mathrm{F}\mathrm{W}-\mathrm{D}\mathrm{W})}{(\mathrm{T}\mathrm{W}-\mathrm{D}\mathrm{W})}\times100$$

#### Leaf Membrane Stability Index (MSI)

Fully expanded fresh leaves were collected from both levels of salinity-treated plants after four weeks of salt application. Using a metal form, the leaves were cut into discs of uniform size, each of 0.1 g, then transferred into test tubes containing 10 ml of dd-H_2_O. The tubes were divided into two sets, the first set was incubated for 30 min in a water bath at 40 °C, while the second set was incubated in boiling water at 100 °C for 15 min (Sairam 1994). The leaf electric conductivity at 40° (C1) and 100 °C (C2) was measured by a conductivity meter, and MSI was calculated as follows:

$$\:\mathrm{M}\mathrm{S}\mathrm{I}=\left(1-\frac{\mathrm{C}1}{\mathrm{C}2}\right)\times100$$

#### Leaf electrolyte leakage (EL)

Leaf discs (0.5 cm diameter each) of fully expanded fresh leaves collected after four weeks of stress imposition from control and salinity-treated plants were cut into uniform sizes, then taken to test tubes containing 15 mL of deionized water with continuous shaking, and immediately, EL in the solution was measured (C0) (Muhammad et al. 2018). The solution EL was measured again after 3 h of incubation at room temperature using a conductivity meter (C3). Then, the test tubes with leaf samples and dd-H₂O were boiled for 10 min, and the electrolyte concentration was measured (TC). The percentage of EL was calculated as follows:

$$\%\:EL=100\:\times\:\frac{(C3-CO)}{TC}$$

#### Stomatal Conductance (SC)

Stomatal conductance was determined from fully developed leaves for plants of both S1 and S2 treatments after the completion of salinity stress, during the day (9:00–10:00 am) with a leaf porometer (Model: SC-1, Decagon Devices, Pullman, WA, USA).

### Fruit yield and quality parameters

Fruits of all treatments were harvested weekly, and the number and weight (g) of fruits/plant were recorded. At the end of the season, the total number and weight of fruit/plant were calculated. Average fruit weight, number, and total weight per plant were measured by adding the number and weight of harvested fruits plant^− 1^ over the season.

#### Fruit firmness

Fruit firmness was measured using 5 representative mature fruits per treatment per replicate by a digital basic force gauge, model BFG 50 N (Mecmesin, Sterling, Virginia, USA).

#### Total dissolved solids (TSS%), titratable acidity (TA%) and vitamin C concentration

For measuring TSS (%), TA (%), and Vitamin C (mg.100 g^− 1^ FW), a representative sample of fruits for each treatment was selected, then washed with dd-H_2_O and air dried at room temperature. After that, the seeds were removed, and the remaining pulp was mixed to extract juice. The juice of each sample was stored at 4 °C until use. Total dissolved solids (TSS%) was measured using a digital refractometer (Pocket Refractometer PAL 3, ATAGO, Japan). Vitamin C concentration (mg.100 g^− 1^ FW) was determined by the oxidation of ascorbic acid with 2,6-dichlorophenol indophenol dye as described by Bajaj and Kaur (1981). TA% of juice was measured using an automatic titrator (HI 902, HANNA Instrument, USA) after the fruit juices were diluted at a ratio of 1:2 juice: dd-H_2_O (Seyoum et al. 2011).

#### Fruit pigments

For measuring fruit pigments, 1 g of tomato fruit was homogenized in 10–20 ml acetone–hexane solvent (1:2 v/v), and levels of lycopene and β-carotene in the supernatant were quantified using the spectrophotometric method described by Nagata and Yamashita (1992).

### Leaf proline, phenols, flavonoids, and free radical scavenging capacity

#### Leaf proline concentration

Proline concentration of fully expanded leaves collected from stressed and non-stressed control plants just after the finishing of the fourth week of salinity application was measured by a scaled-down version of the acid-ninhydrin method of Bates et al. (1973). Half (0.5) g of leaf was homogenized in 10 ml of 3% aqueous sulfosalicylic acid and then filtered with filter papers. Two ml of filtrate was transferred to test tubes containing 2 ml of acid ninhydrin and 2 ml of glacial acetic acid and then kept in a water bath at 100 °C for 1 h, where the reaction was terminated by submerging the test tubes in an ice bath. The reaction mixture was extracted with 4 ml of toluene, and after that, the absorbance was read at 520 nm using a toluene blank. The concentration of proline was estimated by referring to a standard curve of proline and expressed in µg.g^− 1^ FW.

#### Total Phenol Concentration (TPC)

Total phenol concentration (TPC) was measured according to Hoff and Singleton (1977) with some modifications as detailed in Awad et al. 2017. Fifty (50) µl of leaf methanol extract was mixed with 100 µl Folin-Ciocalteu reagent and 850 µl of methanol to be incubated for 5 min at room temperature. The mixture was reacted for 30 min with 500 µl of 20% sodium carbonate, and the absorbance was measured at 750 nm. Total phenolic concentration was expressed as mg gallic acid equivalents per gram of sample (GAE mg. g^− 1^) and the unit was mg gallic acid^−1^g^− 1^ FW.

#### Total Flavonoid Concentration (TFC)

Total flavonoid concentration (TFC) was measured as per the method of Zhishen et al. (1999). A 100 µl of fully expanded leaf extract was added to 4 ml of distilled water, 0.3 ml 5% sodium nitrite, and incubated at room temperature for 5 min. 0.3 ml of 10% aluminium chloride was added and incubated for 6 min; before that, 2 ml of 1 M sodium hydroxide was added. The mixture was immediately diluted with 3.3 ml of distilled water and mixed thoroughly, and absorbance was recorded at 510 nm. Catechin was used as a standard for the calibration curve. The total flavonoid concentration of the extract was expressed as mg catechin equivalents per gram of sample (mg. g^− 1^). So, the TFC concentration was expressed in mg catechin^−1^g^− 1^ FW.

#### DPPH Free Radical Scavenging Activity (FRSC)

FRSC of leaf and fruit samples was quantified in diphenyl-picrylhydrazyl (DPPH) assay according to Boo et al. 2012. Five (5.0) ml of 80 mM DPPH radical solution was added to 1 ml of the leaf extract solution. The reaction was incubated for 30 min, and absorbance was taken at 515 nm using a spectrophotometer. The concentration of sample required to scavenge 50% of DPPH free radical (IC_50_ value) was calculated from the plotted graph of radical scavenging activity against the concentration of extracts. The extraction of protein was performed according to the procedure of Antoniou et al. 2020. The protein quantification was done following the techniques of Bradford (1976).

### Determination of antioxidant enzyme activities

#### Sample preparation

Leaf samples were collected from fully expanded leaves of grafted, self-grafted, and non-grafted plants just after the completion of the 4th week of salinity treatments. Fresh leaf (0.2 g) was homogenized in phosphate buffer (50 mM, pH 7.8) in a pre-chilled mortar, centrifuged (12,000 × *g* at 4 °C) for 20 min, and then the samples were stored at − 80 °C for further analysis.

#### Polyphenol oxidase (PPO; EC 1.14.18.1)

PPO; EC 1.14.18.1 enzyme activity was quantified by catechol oxidation following the methods of Awad et al. (2017). PPO activity was recorded as the changes in the absorbance rate at 430 nm at 1 min and 10 s intervals using a spectrophotometer in a kinetic state. Enzyme activity was recorded as a change in absorbance per min. mg protein (Moghaddam et al. 2019). The PPO activity was measured in unit.min-^1^.g^− 1^ FW.

#### Peroxidase (POD; EC 1.11.1.7)

POD; EC 1.11.1.7 enzyme activity was determined from increased absorbance at 470 nm for 3 min (Castillo et al. 1984). POD enzyme activity was calculated using the extinction coefficient of guaiacol (26.6 mM^− 1^ cm^− 1^), and a complete reaction mixture without H_2_O_2_ was used as a blank. POD activity was expressed in unit.min^− 1^.g^− 1^ FW.

#### Catalase (CAT; EC1.11.1.6)

CAT; EC 1.11.1.6 activity was determined by increased absorbance of carbonate-cobaltate (III) complex ([Co (CO_3_)_3_]Co) after the consumption of H_2_O_2_ at 440 nm for 10 min (Hadwan 2018). CAT enzyme activity was calculated by subtracting the measured absorbance from the absorbance of the solution without enzyme extract. The enzymatic activity of CAT was measured in EU.mg^− 1^ protein.

#### Superoxide dismutase (SOD; EC1.15.1.1)

The SOD; EC 1.15.1.1 activity was determined by monitoring the inhibition of photochemical reduction of nitro-blue tetrazolium chloride at 560 nm according to the methods of Giannopolitis and Ries (1977). SOD enzyme activity was measured in EU.mg^− 1^ protein.

### Data analysis

Analysis of variance (ANOVA) of the recorded data was performed according to Steel and Torrie (1986) to observe important variations among the genotypes under salinity stress using Statistix10 software (McGraw-Hill 2008) and the treatment means were compared using Tukey’s HSD (Honestly Significant Difference) all-pairwise comparisons at the *p* = 0.05 level as a post-hoc test. The traits with considerable variations for graft/genotype versus salt-stress interactions were used for principal component analysis (PCA). PCA and cluster analysis were performed with the software R, version 4.3.3 (Team 2020). A graphical representation of the studied traits was devised with the GraphPad Prism 10.0 program (Prism 2018).

## Results

### Leaf photosynthetic pigments, physiological traits, and inorganic ions concentration

**Fig. 1:**
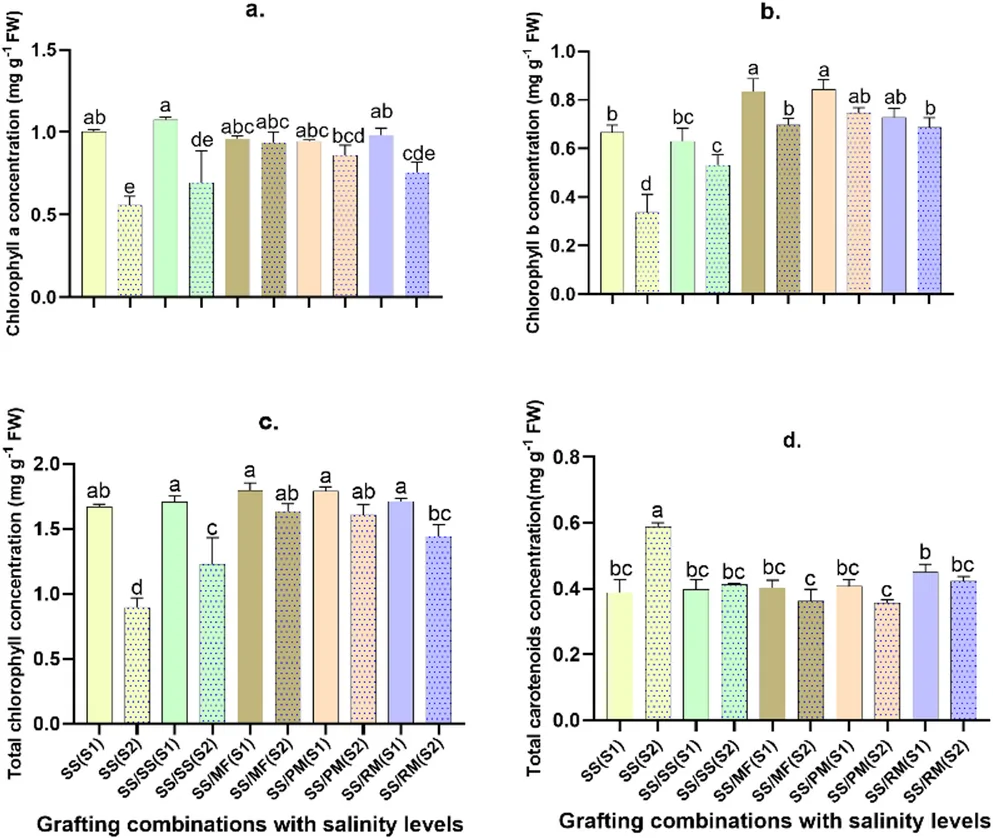
Photosynthetic pigments for non-grafted (SS), self-grafted (SS/SS) and grafted tomato combinations (SS/MF, SS/PM and SS/RM) under low (S1) and high salt (S2) concentrations at greenhouse conditions: (**a**) Chlorophyll a concentration in mg.g^−1^of fresh weight (FW), (**b**) Chlorophyll b concentration (mg.g^− 1^ FW), (**c**) total chlorophyll(mg.g^− 1^ FW), and (**d**) total carotenoids (mg.g^− 1^ FW). Mean ± standard deviation (SD), *n* = 3 with three replicates, and bars with similar letters are statistically identical with *P* ≤ 0.05

FigurePhotosynthetic pigments (chlorophyll a, chlorophyll b, and carotenoids) in leaves of all grafting combinations were highly affected by salt stress. Under low salinity (S1), the leaves of all tested combinations possessed high Chl-a, Chl-b, and total chlorophyll and carotenoids concentration, with variation between combinations SS/SS and SS/RM (Chl-a), SS/MF and SS/PM (Chl-b and total chlorophyll), and SS/RM for total carotenoids (Fig. 1). Increasing water salinity significantly reduced the concentrations of Chl-a, Chl-b, and total chlorophyll in leaves in all combinations, with prominent reduction for nongrafted (SS) and self-grafted plants (SS/SS). Cross-grafted plants (SS/MF, SS/PM, SS/RM) showed higher Chl-a, Chl-b and total chlorophyll leaf concentration under high salt as compared to SS and SS/SS combinations. Non-grafted plants showed the highest total carotenoid concentration under salt stress (Fig. 1).

**Fig. 2:**
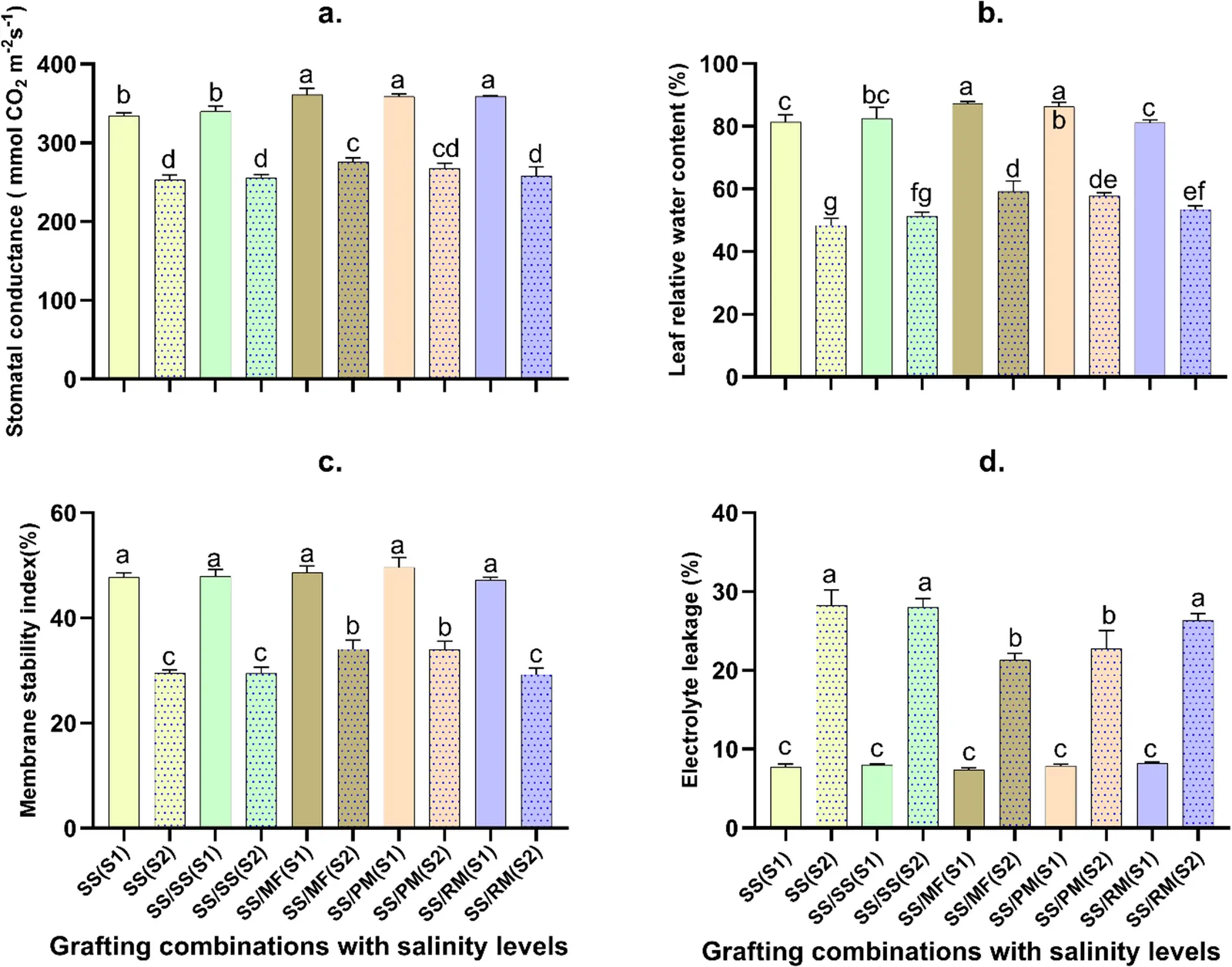
Mean values for leaf physiological traits of non-grafted (SS), self-grafted (SS/SS) and grafted tomato combinations (SS/MF, SS/PM and SS/RM) under low (S1) and high salt (S2) concentrations at greenhouse conditions: (**a**) Stomatal conductance (mmol CO_2_ .m^−2^s^− 1^), (**b**) leaf relative water content (%), (**c**) Membrane stability index (%), and (**d**) leaf electrolyte leakage (%). Mean ± standard deviation (SD), *n* = 3 with three replicates, and bars with similar letters are statistically identical with *P* ≤ 0.05

Leaf physiological parameters were enhanced by grafting on the rootstocks MF, PM, and RM under low and high irrigation water salinity. Higher stomatal conductance (SC) and leaf relative water concentrations (LRWC) was observed for grafting combinations SS/MF and SS/PM under low and high salinity as compared to SS, SS/SS and SS/RM (Fig. 2). Under low water salinity (S1), all tested combinations showed similar positive responses regarding to leaf electrolyte leakage (EL) and enhanced values of membrane stability index (MSI). However, grafting combinations SS/MF and SS/PM recorded the highest MSI under high salt stress (S2). Enhanced leaf EL values were observed with the grafting combination SS/RM, where the differences were not significant from SS and SS/SS tomato combinations (Fig. 2).

**Fig. 3:**
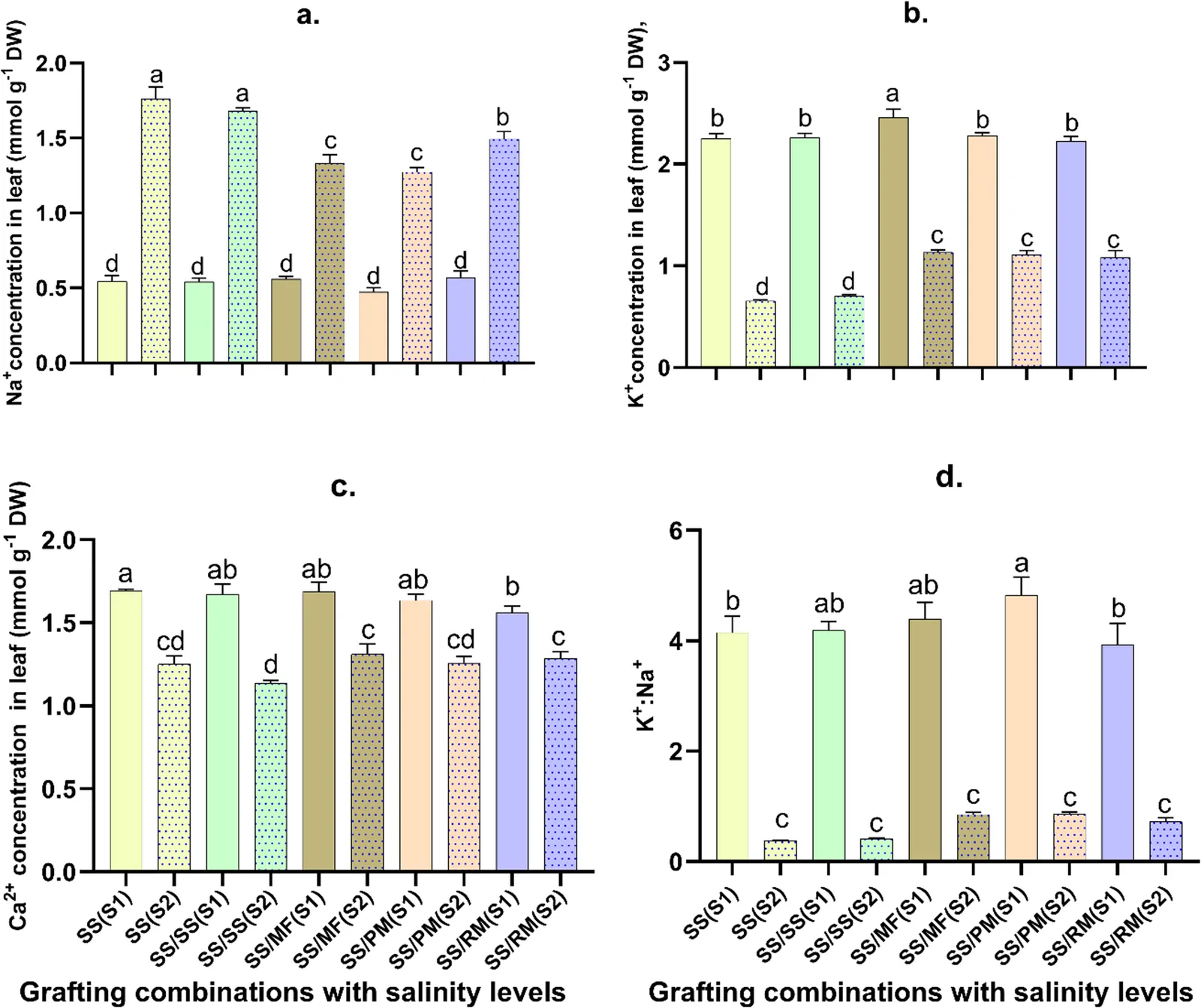
Mean values for leaf ion concentration of non-grafted (SS), self-grafted (SS/SS) and grafted tomato combinations (SS/MF, SS/PM, and SS/RM) under low (S1) and high salt (S2) concentrations in greenhouse conditions: (**a**) leaf Na^+^ concentration in mmolg^−1^of dry weight (DW), (**b**) K^+^ concentration (mmolg^− 1^ DW, c) Ca^+^ concentration (mmolg^− 1^ DW) d) K^+^:Na^+^. Mean ± standard deviation (SD), *n* = 3 with three replicates, and bars with similar letters are statistically identical with *P* ≤ 0.05

**Fig. 4:**
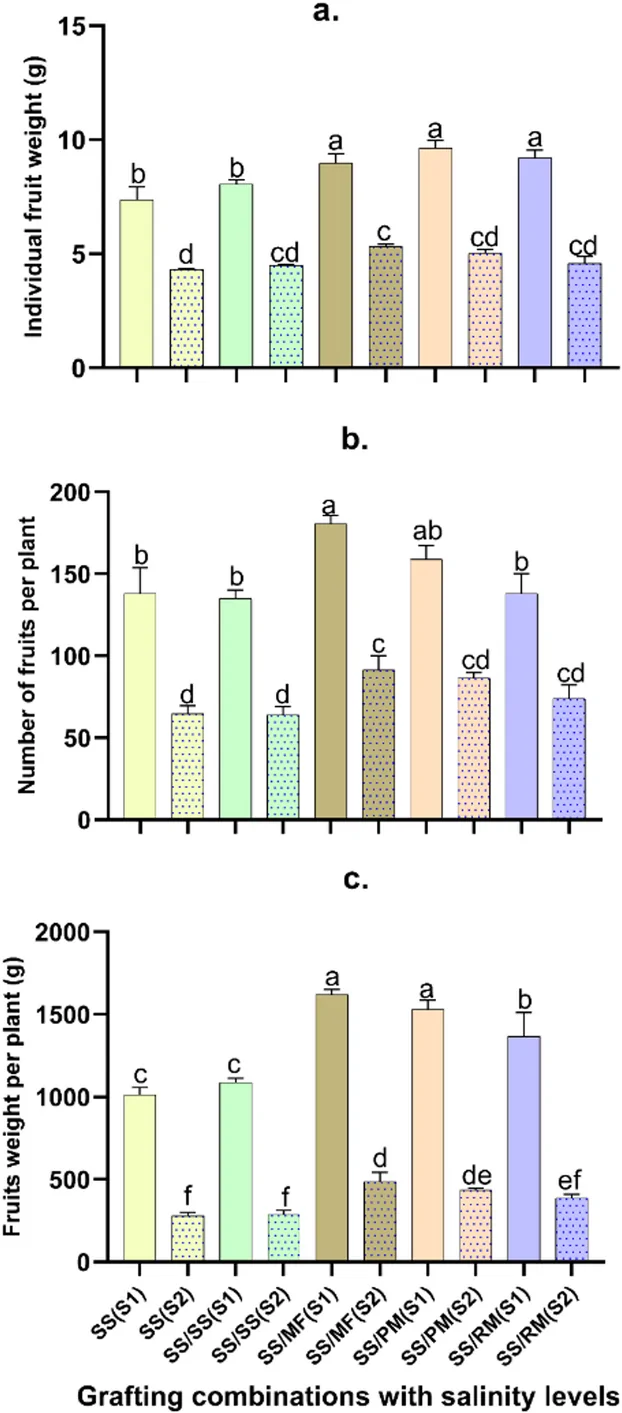
Mean values for fruit parameters of non-grafted (SS), self-grafted (SS/SS) and grafted tomato combinations (SS/MF, SS/PM and SS/RM) under low (S1) and high salt (S2) concentrations at greenhouse conditions: (**a**) weight of individual fruit (g), (**b**) the number of fruitsplant^− 1^, and (**c**) weight of fruitsplant^− 1^ (g). Mean ± standard deviation (SD), *n* = 3 with three replicates, and bars with similar letters are statistically identical with *P* ≤ 0.05

The responses of tomato grafting combinations to salt stress, as indicated by leaf concentrations of inorganic ions (Na^+^, K^+^, Ca^2+^_,_ and K^+^:Na^+^) varied. As shown in Fig. 4, low Na^+^ and high K^+^ and Ca^2+^ leaf concentration were observed for all combinations at low water salinity (S1), with SS/PM showing the highest (4.83) in leaf K^+^:Na^+^ compared with other combinations. Under the high level of water salinity (150 mM of NaCl), the non-grafted and self-grafted plants accumulated the high Na^+^ and the lowest level of K^+^ and Ca^2+^, while the grafting combinations SS/MF, SS/PM and SS/RM slightly reduced Na^+^ and enhanced K^+^ and Ca^2+^ accumulations in plant leaves with K^+^:Na^+^ ratio of 0.85, 0.87 and 0.75, respectively (Fig. 3).

### Fruits yield and quality

The number and weight of harvested fruit plant^− 1^ and the average weight of individual fruit varied due to grafting and irrigation water salinity. The yield and yield attributes parameters of the tomato F_1_ hybrid ‘Super sweet’ (SS) grafted on the rootstocks MF (SS/MF), PM (SS/PM), and RM (SS/RM) increased at low levels of irrigation water salinity (18.2 mM of NaCl) (Fig. 4). A reduction in the number and weight of fruits per plant and the average weight of individual fruit was observed in all tested combinations under the high level of irrigation water salinity (150 mM of NaCl). The grafted combination SS/MF, SS/PM, and SS/RM produced the higher fruit number, weight per plant, and average individual fruit weight compared to the non-grafted (SS) and self-grafted (SS/SS) plants (Fig. 4). A significant increase was observed in SS/MF for fruit plant^− 1^ (91.41), fruit yield plant^− 1^ (488.1 g), and the average weight of individual fruit (5.33 g), followed by SS/PM with 86.53 fruits plant^− 1^, fruit yield of 435.40 g, and 5.03 g per individual fruit, where SS/RM produced 73.99 fruits plant^− 1^, 339.57 g. plant^− 1^ and 4.59 g. individual fruit^− 1^ (Fig. 4).

**Fig. 5:**
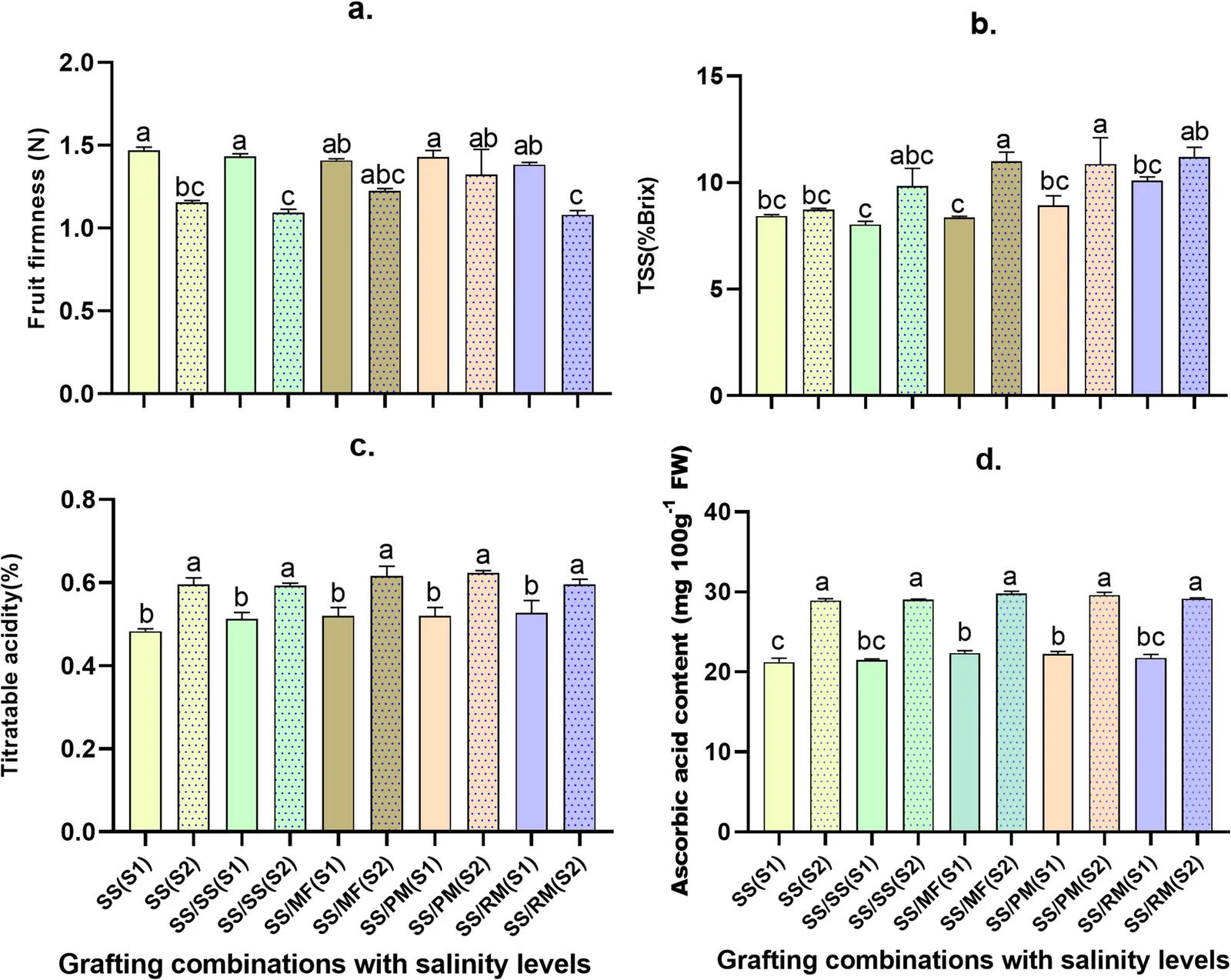
Mean values for fruit quality parameters of non-grafted (SS), self-grafted (SS/SS) and grafted tomato combinations (SS/MF, SS/PM and SS/RM) under low (S1) and high salt (S2) concentrations at greenhouse conditions: (**a**) Fruit firmness (N), (**b**) TSS (%Brix), (**c**) titratable acidity (%), and c) ascorbic acid concentrations (mg.100 g^− 1^ FW). Mean ± standard deviation (SD), *n* = 3 with three replicates, and bars with similar letters are statistically identical with *P* ≤ 0.05

**Fig. 6:**
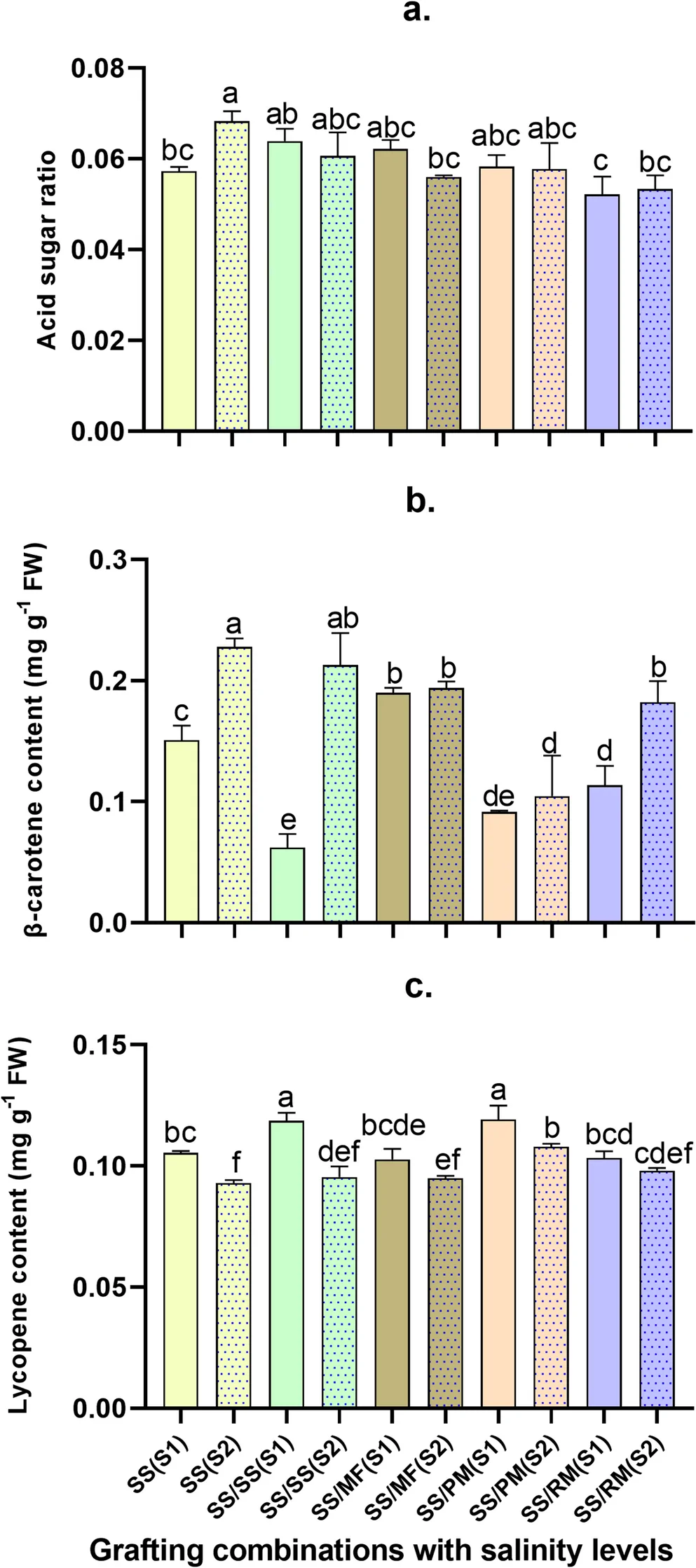
Mean values of fruit’s (**a**) acid sugar ratio, (**b**) ß-carotene concentrations (mg.g^− 1^ FW), and (**c**) Lycopene concentrations (mg.g^− 1^ FW) for non-grafted (SS), self-grafted (SS/SS) and grafted tomato combinations (SS/MF, SS/PM and SS/RM) under low (S1) and high salt (S2) concentrations at greenhouse conditions. Mean ± standard deviation (SD), *n* = 3 with three replicates, and bars with similar letters are statistically identical with *P* ≤ 0.05

High water salinity (S2) increased fruit vitamin C concentrations (mg.100 g^− 1^ FW) and titratable acidity (%) of all plants when compared to low water salinity (S1) (Fig. 5). Fruit TSS (%) was increased under high salt stress by grafting on the rootstocks MF and PM. Fruits with greater firmness were observed for the combinations SS, SS/SS, and SS/PM at low water salinity. The acid sugar ratio was found to be higher in the S1, and S2 treatments for the non-grafted and the self-grafted plants. We observed enhanced lycopene concentrations in the fruits for tomato grafts SS/SS at S1 and lower for SS/MF at both levels of water salinity (S1 and S2), and high ß-carotene concentration in the fruits was observed for non-grafted tomato (SS) (Fig. 6).

### Leaf proline, phenolic compounds and antioxidant capacity

#### Proline

**Fig. 7:**
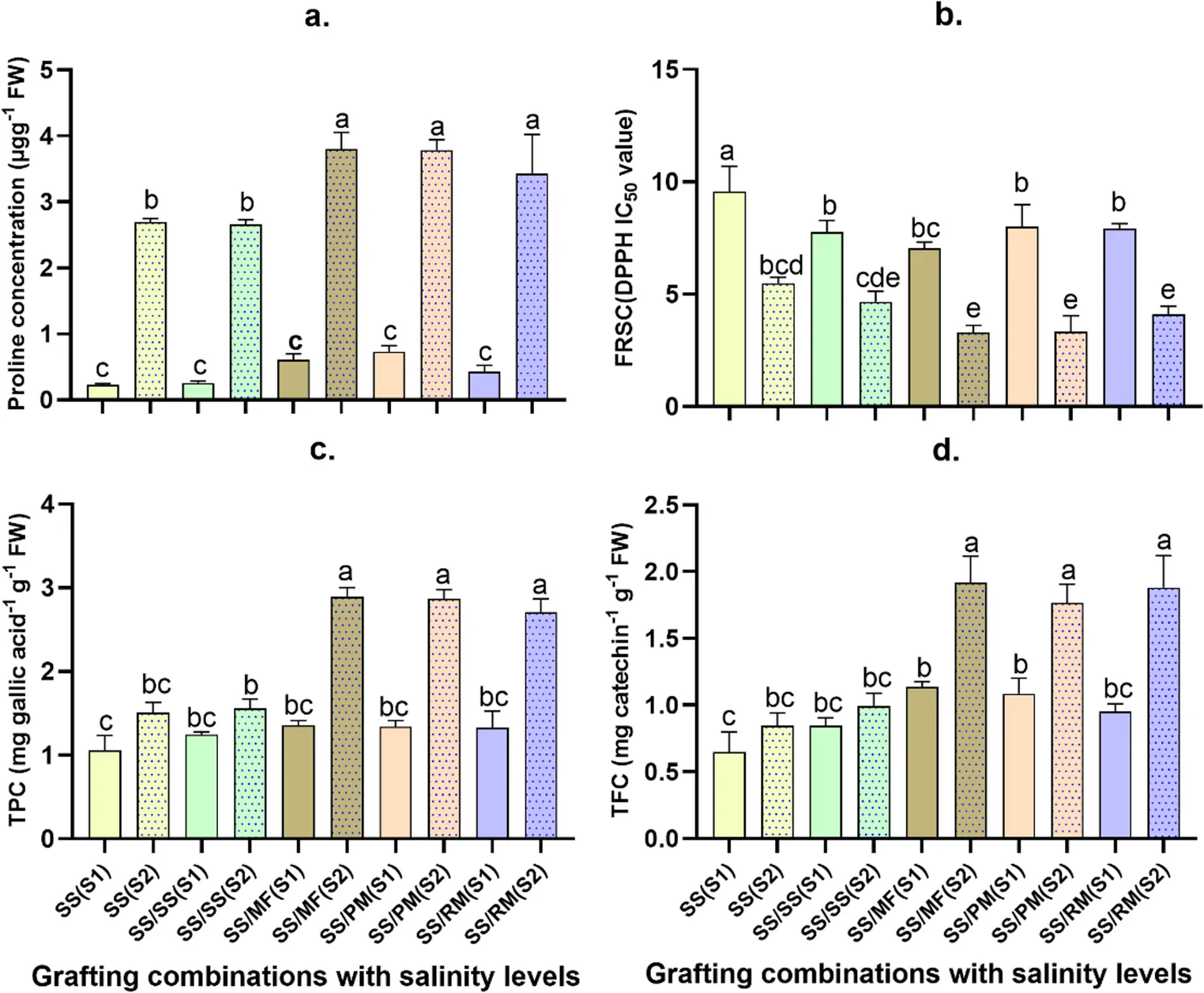
Biochemical analysis of leaves of nongrafted (SS), self-grafted (SS/SS) and grafted (SS/MF, SS/PM and SS/RM) as affected by low (S1) and high (S2) levels of irrigation water salinity at greenhouse conditions: (**a**) Proline concentrations (µgg^− 1^ FW), (**b**) Free radical scavenging capacity (DPPH, IC_50_ values), c)Total phenolic compounds (TPC) in mg gallic acid^−1^g^− 1^ FW, and d) Total flavonoids compounds (TFC, mg catechin^−1^g^− 1^ FW). Mean ± standard deviation (SD), *n* = 3 with three replicates, and bars with similar letters are statistically identical with *P* ≤ 0.05

Higher proline concentration was measured in leaves of grafted plants compared to nongrafted and self-grafted combinations. The grafting combinations SS/MF, SS/PM, and SS/RM showed enhanced proline accumulation in leaves under high salt, with values of 3.79, 3.78, and 3.43 µg.g^− 1^ FW, as compared to 2.69 and 2.66 µg.g^− 1^ FW for SS and SS/SS (Fig. 7). Grafting on MF, PM, and RM rootstocks increased the proline concentration of leaves under low salt, but the proline values were lower than those observed under high salt stress (Fig. 7).

#### Polyphenolic compounds (TPC and TFC)

Leaf TPC and TFC were significantly increased under high salt treatment when grafted on MF, PM, and RM rootstocks. The leaves contained 2.89, 2.87, and 2.70 µg.g^− 1^ FW TPC and 1.92, 1.88, and 1.72 µg.g^− 1^ FW TFC for grafting combinations SS/MF, SS/PM, and SS/RM, respectively (Fig. 7). Under low salt, non-grafted (SS) plants accumulated the least TPC and TFC with 1.06 and 0.65 µg.g^− 1^ FW.

#### Antioxidant enzyme system

Decreased free radical scavenging capacity (FRSC) in leaves was observed under high salt treatment of all grafting combinations. Leaves of the grafted tomato (SS/MF) attained the lowest (3.29 µg.g^− 1^ FW) concentration of FRSC under high salt stress, followed by SS/PM (3.33 µg.g^− 1^ FW) and SS/RM (4.10 µg.g^− 1^ FW), respectively. Non-grafted (SS) showed the maximum FRSC (9.59 µg.g^− 1^ FW) under a low level of water salinity (Fig. 7).

**Fig. 8:**
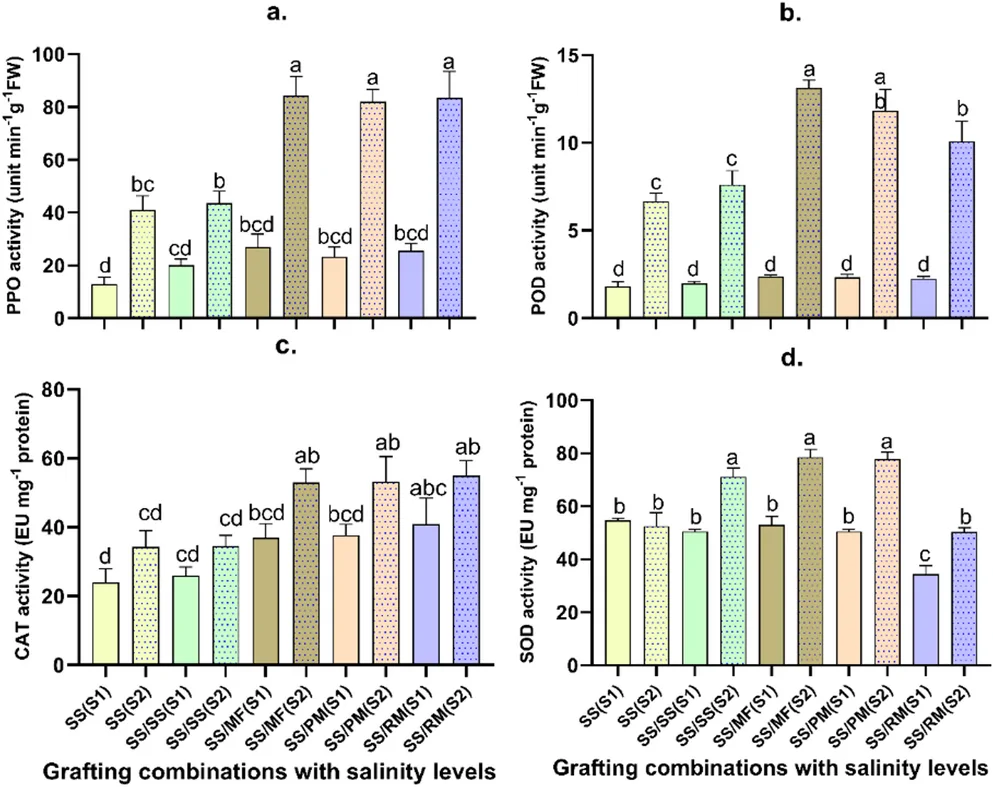
Biochemical analysis of leaves of nongrafted (SS), self-grafted (SS/SS) and grafted (SS/MF, SS/PM and SS/RM) as affected by low (S1) and high (S2) levels of irrigation water salinity at greenhouse conditions: (**a**) PPO: Polyphenol oxidase (PPO; EC 1.14.18.1) enzyme activity (unit.min^− 1^.g^− 1^ FW), (**b**) POD: Peroxidase (POD; EC 1.11.1.7) enzyme activity (unit.min^− 1^.g^− 1^ FW), (**c**) CAT: Catalase (CAT; EC 1.11.1.6) enzyme activity (EU.mg^− 1^ protein), and (**d**) SOD: Superoxide dismutase (SOD; EC 1.15.1.1) enzyme activity (EU.mg^− 1^ protein). Mean ± standard deviation (SD), *n* = 3 with three replicates, and bars with similar letters are statistically identical with *P* ≤ 0.05

Leaf PPO and CAT enzymes activities increased under high salt treatment for the grafting combinations SS/MF (84.25 and 52.99 unit min^− 1^.g^− 1^ FW), SS/PM (82.04 and 53.15 unit min^− 1^.g^−1^FW), and SS/RM (83.42 and 54.92 unit min^− 1^.g^− 1^ FW). The minimum CAT and PPO activity was measured in the leaves of the non-grafted tomato at low and high salt stress (Fig. 8a & b). The highest enzymatic activity of POD was measured in the leaves of the grafted tomato SS/MF (13.14 unit min^− 1^.g^− 1^ FW) under high salt treatment, while SS and SS/SS recorded the minimum level of POD in the leaves (Fig. 8c). For leaf SOD enzyme activity, SS/SS, SS/MF, and SS/PM showed higher SOD activity in the leaves under high water salinity. All tested combinations showed decreased levels of SOD activity, and we recorded for SS/MF, SS/PM, and SS/RM 78.53, 77.76, and 50.29 unit min^− 1^.g^− 1^ FW, respectively, under a low salinity level. At higher salinity stress (S2), we interestingly found higher enzymatic activities of SOD for the self-grafted (SS/SS) plants (Fig. 8).

### Principal component analysis (PCA) for the measured parameters

**Fig. 9:**
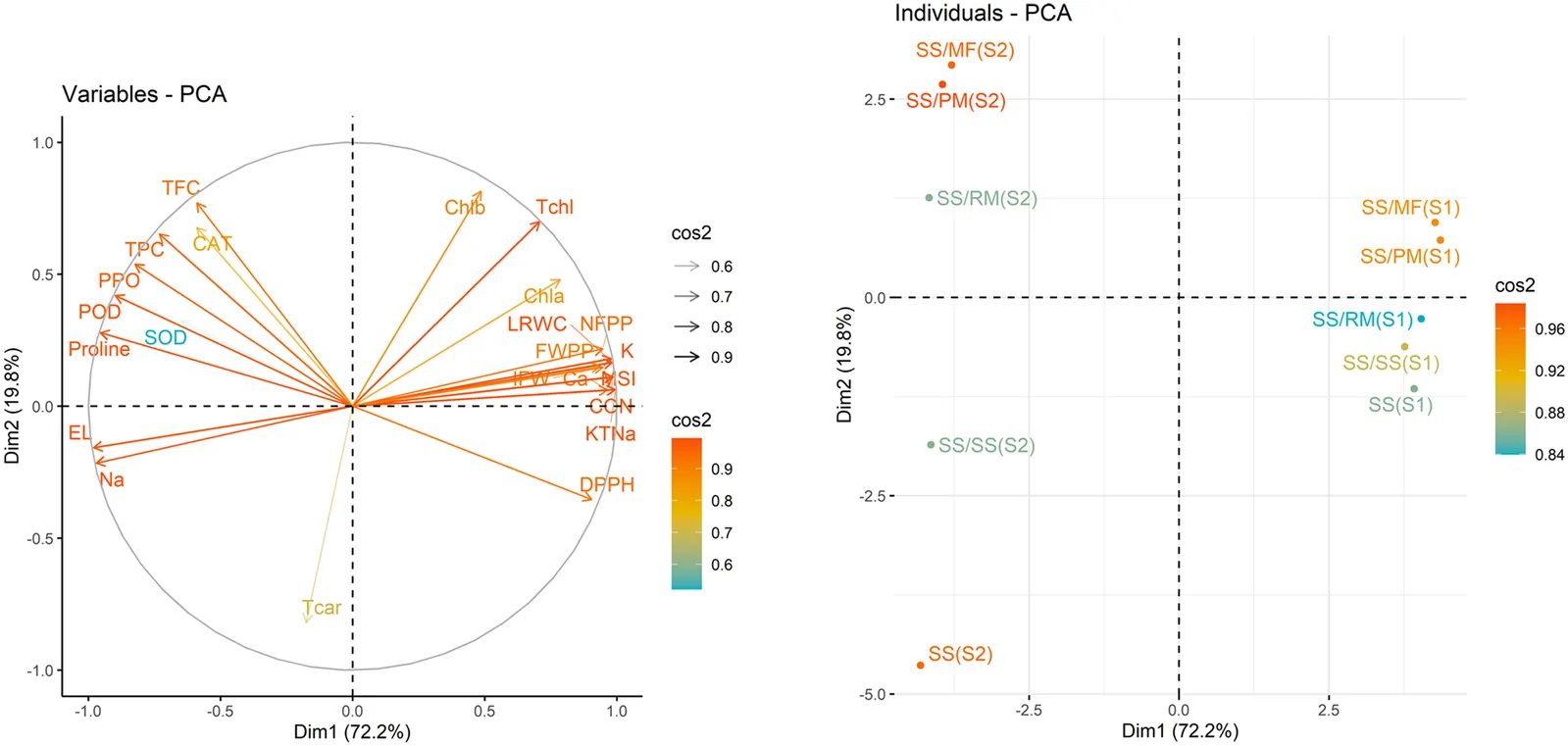
Principal Component Analysis (PCA) of nongrafted (SS), self-grafted (SS/SS) and grafted (SS/MF, SS/PM and SS/RM) as affected by low (S1) and high (S2) levels of irrigation water salinity under greenhouse conditions: (**A**) Control (S1: water salinity of 18.2 mM), and (**B**) high salt (S2: 150 mM NaCl in irrigation water) under greenhouse conditions. Here, Chla: leaf chlorophyll a concentration, Chlb: leaf chlorophyll b concentration, CON: leaf stomatal conductance, LRWC: leaf relative water concentration, MSI: membrane stability index of fresh leaves, EL: Electrolyte leakage), leaf ions concentrations (Na: leaf Na^+^ concentration, K: leaf K^+^ concentration, Ca: leaf Ca^2+^ concentration and KTNa: leaf K^+^:Na^+^), yield traits (IFW: individual fruit weight, NFPP: number of fruitsplant^− 1^ and FWPP: fruits weightplant^− 1^), Proline: leaf proline concentration, DPPH: free radical scavenging capacity (FRSC) in DPPH IC_50_, TPC: total phenol concentration, TFC: total flavonoids concentration, PPO: Polyphenol oxidase (PPO; EC 1.14.18.1) enzyme activity, POD: Peroxidase (POD; EC 1.11.1.7) enzyme activity, CAT: Catalase (CAT; EC 1.11.1.6) enzyme activity, SOD: Superoxide dismutase (SOD; EC 1.15.1.1) enzyme activity

**Fig. 10:**
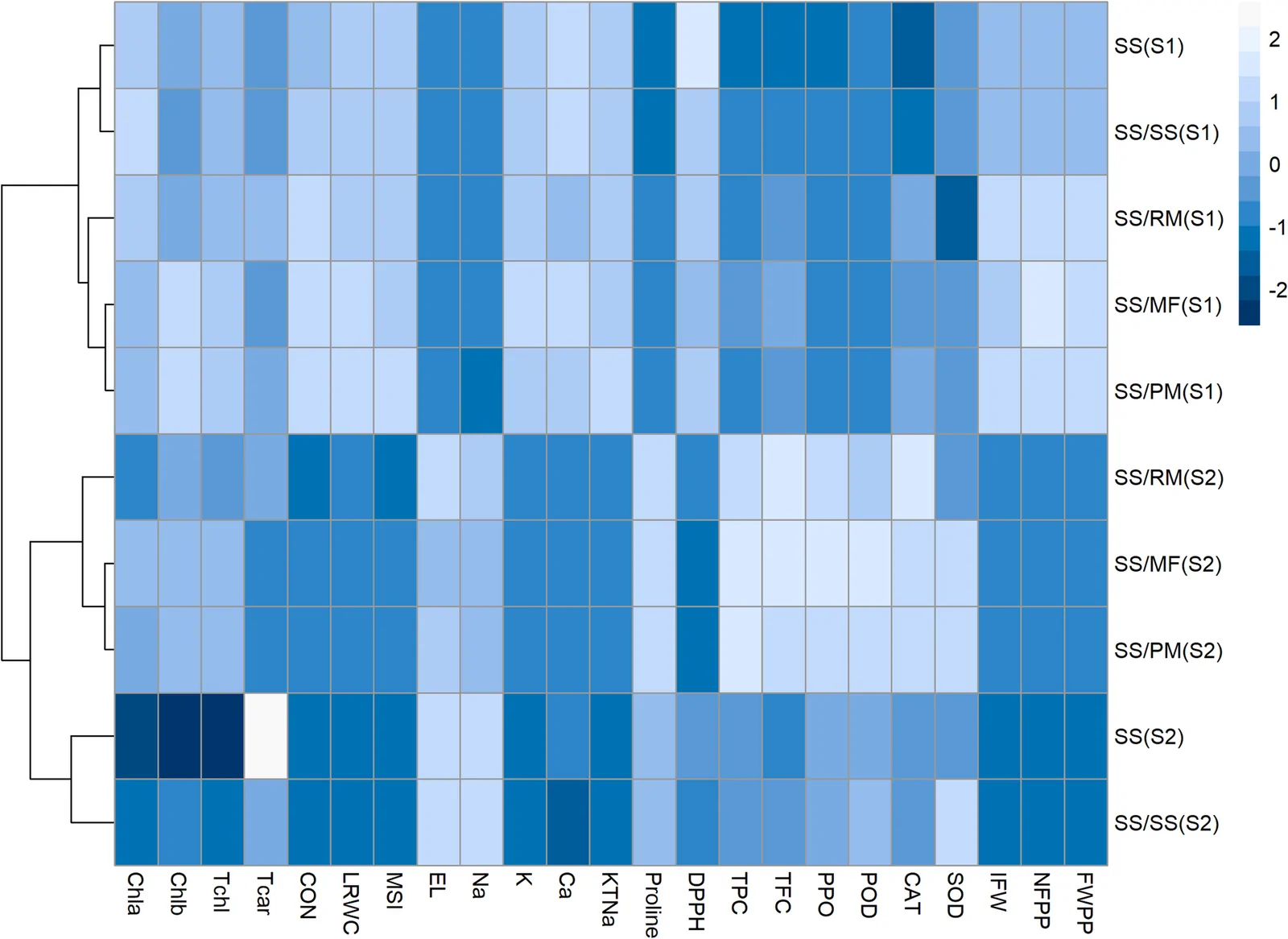
Heatmap clustering of nongrafted (SS), self-grafted (SS/SS) and grafted (SS/MF, SS/PM and SS/RM) as affected by low (S1) and high (S2) levels of irrigation water salinity under greenhouse conditions: (**A**) Control (S1: water salinity of 18.2 mM ), and (**B**) high salt (S2: 150 mM NaCl in irrigation water) under greenhouse conditions. Here, Chla: leaf chlorophyll a concentration, Chlb: leaf chlorophyll b concentration, CON: leaf stomatal conductance, LRWC: leaf relative water content, MSI: membrane stability index of fresh leaves and EL: electrolyte leakage), leaf ions concentrations (Na: leaf Na^+^ concentration, K: leaf K^+^ concentration, Ca: leaf Ca^2+^ concentration and KTNa: leaf K^+^:Na^+^), yield traits (IFW: individual fruit weight, NFPP: number of fruitsplant^− 1^ and FWPP: fruits weightplant^− 1^), Proline: leaf proline concentration, DPPH: free radical scavenging capacity (FRSC) in DPPH IC_50_, TPC: total phenol concentration, TFC: total flavonoids concentration, PPO: Polyphenol oxidase (PPO; EC 1.14.18.1) enzyme activity, POD: Peroxidase (POD; EC 1.11.1.7) enzyme activity, CAT: Catalase (CAT; EC 1.11.1.6) enzyme activity, SOD: Superoxide dismutase (SOD; EC 1.15.1.1) enzyme activity

PCA analysis discriminated the grafting combinations in the PC-biplot according to their responses to low and high salt treatments based on all measured physio-morphological traits, yield, ions concentrations and antioxidant activity system traits (Fig. 9). As shown in the Fig. 10, the dispersion of grafted combination (SS/MF, SS/PM and SS/RM), self-grafted (SS/SS) and nongrafted (SS) revealed the stress responses under the effects of salt treatment. We observe that PC1 and PC2 explained 72.20% and 19.8% of the total variance, respectively, with an accumulative contribution of 93.5% for the total variation of the measured traits. The PC-biplot revealed two distinct groups of studied traits; the first group accommodated all the physiological (except EL), ionic (except Na^+^) and yield attribute traits of grafted (SS/MF, SS/PM and SS/RM), self-grafted (SS/SS) and non-grafted (SS) combinations under low salt stresses (S1) (Fig. 9). In the second group, Na^+^, El and almost all the biochemical traits (SOD, Proline, POD, PPO, TPC, CAT and TFC) were plotted. We observed that Na^+^, EL, and other stress-related biomolecules were close to each other, which revealed their strong positive correlations. The results showed that the grafted combinations SS/MF, SS/PM, and SS/RM were very close to each other in the first and second quartiles of the biplot under low (S1) and high (S2) salt treatments. Moreover, the self-grafted (SS/SS) and non-grafted (SS) combinations were plotted in the third and fourth quartiles of the biplot under low (S1) and high (S2) salt treatments.

The heatmap clustering represented the analysed traits’ mean values for all of the grafting combinations for both control (S1) and salinity (S2) treatments (Fig. 10). It was clear that the grafting combinations were clustered in two main clusters for the S1 and S2, which revealed the significant effect of salinity. The grafting combinations were clustered according to their similarities in responses to salinity levels.

In the heatmap (Fig. 10), darker colours represented lower mean values of the respective physio-morphological and biochemical characters. Both in control (S1) and salinity (S2) conditions, grafting combinations SS/MF, SS/PM, and SS/RM showed higher values for almost all studied traits in comparison with non-grafted SS and self-grafted SS/SS. For this reason, these combinations were grouped in the same sub-clusters for both control (S1) and salinity (S2) treatments. We observed that under salinity treatment (S2), SS/MF, SS/PM, and SS/RM were grouped in the same subcluster. Similarly, in control (S1) conditions, these combinations were accommodated in one sub-cluster. Non-grafted SS and self-grafted SS/SS were placed in the same subclusters for S1 and S2, respectively. In the study, the grafting combinations SS/MF, SS/PM, and SS/RM showed higher values for physiological traits except EL, ion concentrations except Na, and fruit yield traits (IFW, NFPP, and FWPP) in S1. Again, higher responses for proline, TPC, TFC, PPO, POD, CAT, and SOD under salinity (S2) were recorded for SS/MF, followed by SS/PM and SS/RM. In salinity stress (S2) higher values for EL and Na were observed for SS and SS/SS.

## Discussion

Soil salinity is an increasing global concern driven by climate change and anthropogenic activities (FAO, 2022). Globally, about 53% of the agricultural lands (20% cultivated and 33% irrigated lands) suffered from high salinity (Shrivastava and Kumar 2015; FAO, 2022). Elevated salinity exerts detrimental effects on almost all plant morpho-physiological, biochemical, and cellular processes, mainly photosynthesis, transpiration, and photorespiration (Hniličková et al. 2019; Ibrahimova et al. 2019; Pailles et al. 2020; Ur Rahman et al. 2021). Consequently, plant growth and development are impaired, resulting in reduced crop yield (Munns and Tester 2008; Kumar et al. 2021; El-Banna et al. 2022).

Tomato is documented to have intraspecific and interspecific variations in salt tolerance (Akinci et al. 2004; Pailles et al. 2017). Under salinity stress, tomato plants undergo alterations in many physio-morphological traits such as root and shoot growth and dry matter accumulation, ion transport mechanisms, and the photosynthetic activities (Dasgan et al. 2002; Alam et al. 2021; Bonarota et al. 2022). Furthermore, these responses to salt stress vary depending on the genetic potential of the genotypes to combat the harmful effects of ionic toxicity (Raza et al. 2017; Borbély et al. 2020; Alam et al. 2021).

Vigorous rootstocks with well-developed root systems could enhance the growth, nutrient uptake, yield, and physiological activities under salinity stress. Their deeper and more extensive root architecture, coupled with increased root biomass, enables the plants to maintain better root hydraulic conductivity. This allows stable water uptake to the plants and improved osmotic adjustment mechanism to minimize the detrimental effect of salinity (Aydın, 2024; Martínez et al. 2024). Grafting on resistant rootstocks was found to enhance salinity tolerance in tomato by restricting the entrance of toxic salt ions (Na^+^, Cl^−^) and their further transport to the shoot and leaves. More precisely, they increase the selective absorption of beneficial K^+^ and Ca^2+^ and improve the ionic balance, such as higher K^+^/ Na^+^ in the plant tissue (Estañ et al. 2005; Martinez-Rodriguez et al. 2008; Reham Abdalla 2017; Aydin 2024).

. In other grafting experiments (Martinez-Rodriguez et al. 2008) with a salt excluder rootstock ‘Radja’ and an includer rootstock ‘Pera’ with the scion ‘Moneymaker’ under 150 mM NaCl salt stress, lower leaf Na^+^ concentration and higher root Na^+^ concentration than the self-grafted ‘Moneymaker’. demonstrating that ‘Moneymaker’ was less capable of accommodating Na^+^ in roots when self-grafted than grafted on ‘Radja’ or ‘Pera’ rootstocks (Martinez-Rodriguez et al. 2008). In another report, grafting of ‘Moneymaker’ scion with the ‘Pera’ rootstock performs better in terms of yield, with higher leaf Na^+^ and Cl^−^ accumulation when compared to self-grafted plants. The authors suggested that ‘Pera’ possesses mechanisms to tolerate salinity (Santa-Cruz et al. 2002). Wahb-Allah et al. (2014) conducted a pot experiment in soil media under greenhouse conditions with control (1.2 dS.m^− 1^) and saline (4.5 dS.m^− 1^) treatments with scion ‘Farida’ and rootstock ‘Unifort’. They found that grafting results in higher vegetative growth and fruit yield along with Na^+^ and Cl^−^ ion exclusion. In another grafting study for salinity stress tolerance in a sand tank, rootstock ‘ Maxifort F_1_’ was used for the scion of ‘Big Dena’ at five levels of salinity treatment (1.2 dSm^− 1^, 4.0 dS.m^− 1^, 8.5 dS.m^− 1^, 12 dS.m^− 1^, and 15.8 dS.m^− 1^, respectively). They observed higher absolute yield and higher ion regulation, mainly Na^+^ exclusion, in grafted plants. Semiz and Suarez (2019) reported that ‘ Maxifort F_1_’ was able to reduce the uptake of Na^+^ through the roots and enhance the influx of Ca^2+^ and K^+^. (Semiz and Suarez 2019). Four rootstock QTLs were identified that govern the scion leaf K^+^, Mg^2+^, B^+^, and Mo^2+^. Again, three QTLs of which two were responsible for scion leaf K^+^ and Ca^2+^, were found to control rootstock-mediated solid soluble content transport inside the scion under the effect of moderate salinity (Asins et al. 2015). We also found that the salt-tolerant rootstocks efficiently controlled the influx of Na^+^ and maintained proper cellular K^+^ and Ca^+^ in the plant, consequently reducing leaf Na^+^ concentration by restricting the entrance of harmful ions through the root systems. It was observed that the rootstock Maxifort F1 reduced 23.90% of the leaf Na^+^ accumulation and increased 39.50% of the K^+^ uptake in the tomato plant (Sarkar et al. 2026). Aydin (2024) evaluated the impact of grafting commercial tomato cultivars onto wild *Solanum pimpinellifolium* and *Solanum habrochaites* rootstocks under salt-stress conditions in a hydroponic system. Grafted plants demonstrated improved salt tolerance compared to non-grafted controls, reflected in more vigorous growth and balanced leaf mineral profiles. More precisely, wild rootstocks enhanced nutrient accumulation while limiting the inclusion of sodium and chloride ions, which indicated efficient ion-regulation capacity. The findings suggested that these wild tomato species offer valuable rootstock options for improving salinity tolerance in sustainable tomato production (Aydin 2024).

Similarly, in our experiments, it was found that PimpM021 and Maxifort F_1_ rootstocks were able to reduce the transport of Na^+^ in scions. We observed higher amount leaf chlorophylls, leaf relative water content, and lower membrane damage, along with higher conductance in grafted plants compared to non-grafted plants. Salt-tolerant rootstocks have a deep and branched root structure with stable water and nutrient uptake, with vigorous growth, which results in better management of cell turgor and stability, better stomata conductance, and carbon assimilations (Zhang et al. 2018). Oztekin and Tuzel (2011) examined the effect of grafting on salinity tolerance from 50 to 300 mM NaCl salinity treatments for 15 days in nutrient solution under greenhouse conditions. They found lower leaf damage with higher leaf number and stem thickness under salinity stress in the grafted combinations (Oztekin and Tuzel 2011). Our results were also reported by others who observed that the higher the photosynthetic activities, the higher the physiological activities such as transpiration, leaf conductance, and stability (Orsini et al. 2013; Zhang et al. 2018).

In our study, grafting increased the fruit yield in the control condition in comparison to non-grafted or self-grafted plants. However, under the increased salinity conditions, rootstocks ‘ Maxifort F_1_’ and ‘PimpM021’ showed higher fruit yield as compared to control or non-grafted plants. In a previous study, it was observed that the recombinant inbred lines (RILs) derived from *Solanum pimpinellifolium*, grafted with the commercial tomato hybrid, were efficient enough to increase the fruit yield (Asins et al. 2015). Caradania et al. (2020) conducted a two-year study with a processing-type tomato cultivar ‘TC 266’ grafted on an interspecific rootstock ‘RS01658654’ and reported that grafting increased the morpho-physiological traits with a higher number of flowers and leaves, but marketable fruit yield was increased only for a year. Moreover, grafting with the interspecific rootstock enhanced the number of fruits and fruit dry weight without affecting fruit quality (Caradonia et al. 2020). In previous research, tomato genotype ‘Tamaris’ was grafted onto three rootstocks, ‘Maxifort’, ‘Heman’, and ‘Efial’ under greenhouse conditions. Grafting increased the marketable tomato fruit yield by 30%, but the quality traits, such as ascorbic acid, total phenols, and antioxidant activities, were reduced (Vrcek et al. 2011). However, in another recent study, beyond increasing the yield, the Maxifort F_1_ rootstock improved the fruit quality traits such as ascorbic acid, total phenols, flavonoids, and carotenoids, such as lycopene and β-carotene contents. More interestingly, self-grafted Areenez plants showed moderated salinity stress resilience by producing 7.62% less fruit yield than the Maxifort F_1_ under saline conditions, suggesting self-grafting as a cost-effective technique for tomato production under saline-prone areas (Sarkar et al. 2026). The fruit yield and quality parameters, such as TSS, ascorbic acid, etc., improved in our research under salinity conditions with different grafting rootstocks in comparison with self-grafted or non-grafted plants, which were also in agreement with previous findings (Koleška et al. 2018; Abdelaziz and Abdeldaym 2019; Coban et al. 2020). In another study, it was investigated how different wild and hybrid tomato rootstocks enhance the plant’s ability to cope with salt stress when grafted with a commercial scion after exposing them to saline versus non-saline growing conditions. They recorded growth, physiological parameters, yield, and post-harvest quality traits, and it was concluded that not all rootstocks performed equally under salinity stress. In particular, certain wild-derived hybrids provided the best salt-resilience, making them promising candidates for use in salt-affected areas of tomato production (Zeist et al. 2023).

If the plants are exposed to excess salinity stress, the salt concentration in the plant tissue is also raised to a harmful level, which ultimately generates ROS-associated oxidative damage. To minimize oxidative damage, plants activate enzymatic and non-enzymatic antioxidant defence systems. An effective and efficient antioxidant defence system is found to play a pivotal role in enhanced salinity tolerance in tomato. A salt-tolerant wild tomato has been shown to have increased activities of SOD, CAT, and APX enzymes. A tolerant rootstock efficiently minimizes the ROS generation and resultant oxidative damages to the plant cells and tissues, maintaining cell stability (Shalata et al. 2001; Mittova et al. 2004; Bonarota et al. 2022). Proline, phenols, flavonoids, and antioxidant enzyme activities such as PPO, POD, CAT, and SOD were enhanced in grafted plants under salinity treatments. Higher activities of antioxidant enzymes such as CAT and POD were observed in grafted tomatoes and are proposed to be responsible for the improved salinity tolerance by reducing oxidative damages (Oztekin and Tuzel 2011; Sarkar et al. 2026). At elevated salinity, grafted tomatoes with tolerant rootstocks accumulate higher levels of protective compounds like proline and carbohydrates while minimizing the damage caused by lipid peroxidation (Sarkar et al. 2026). In our study, we observed higher activities of antioxidants in grafted plants, which suggests a role of antioxidant enzymes in reducing oxidative damage due to salinity stress. For the self-grafted (SS/SS) plants, higher enzymatic activity of SOD may contribute to enhanced resilience to salinity stress due to the grafting operation (Sarkar et al. 2026). These findings were also supported by other researchers (He et al. 2009; Sumalan et al. 2020; Aydın, 2024).

PCA allows the identification of plant characters that play a prominent role in response to stress imposition (Raza et al. 2017; Alam et al. 2021). From the PCA biplot, we observed the studied plant traits, different grafting combinations, under two levels of salinity. It was found that salinity exerted a negative impact on the plant physio-biochemical, photosynthetic, and yield traits in all combinations, grafted or non-grafted plants. Salinity stress caused by ionic imbalance, mainly excessive Na^+^ accumulation, decreases photosynthesis and impairs physiological activities. In our study, it was observed that Na^+^ showed a very strong negative correlation with physiological and yield traits, which further demonstrates the harmful effect of salinity on plant growth and yield. The grafting combinations SS/MF, SS/PM, and SS/RM were placed in the first quadrant of the biplot due to their enhanced performance in the higher salinity treatment, though those were also affected by salinity, but to a lesser extent than non-grafted or self-grafted plants. The PCA biplot showed a similar dispersion pattern for the grafted combinations of the studied morpho-physiological and biochemical traits as reported in other studies (Sivakumar et al. 2023; Aydın, 2024).

## Conclusion

The present study highlighted the impact of grafting on the morpho-physiological and biochemical traits, yield, and quality of tomatoes under irrigation with elevated salt stress, which is a major constraint for sustainable tomato production. Tomato grafting on wild or resistant rootstocks could be a promising and sustainable approach to combat salinity stress for desirable yield and quality. The enhanced salt tolerance in the grafted plants was likely due to the rootstocks and scions’ interactions, the rootstock’s ability to exclude harmful Na^+^ from the root or reduce Na^+^ translocation to the scion, as well as their antioxidant defence mechanism. An efficient antioxidant system, because of tolerant rootstocks, enables the plants to maintain proper cellular function and integrity by neutralizing the ROS under the salinity stress. It was observed that beside the commercial rootstock Maxifort F_1_ (MF), the wild rootstock Pimp-M021’(PM) were efficient enough to minimize the harmful effect of salinity stress, confirming its morphological, physiological and biochemical attributes for salt stress resilience. Our findings provide insight into the biochemical mechanism for enhanced salinity tolerance in grafted tomato. Further evaluation of various grafting combinations involving more diverse rootstocks and scions, together with detailed molecular and anatomical analysis should be conducted to improve salinity tolerance in tomato.

## Data Availability

The data presented in this study are available on request from the corresponding authors.
